# Improving Adherence to National Institute for Health and Care Excellence (NICE) Guidelines for the Use of Tranexamic Acid in Primary Total Knee Arthroplasty: A Quality Improvement Initiative

**DOI:** 10.7759/cureus.76111

**Published:** 2024-12-21

**Authors:** Ahmed Hamed, Muhammad A Hamid, Chloe Lawrence, Islam A Sherif, Zubair Younis

**Affiliations:** 1 Trauma and Orthopaedics, University Hospitals Birmingham, Birmingham, GBR; 2 Orthopaedics, The Royal Wolverhampton National Health Service (NHS) Trust, Wolverhampton, GBR

**Keywords:** nice guidelines, quality improvement projects, re-audit, total knee arthroplasty (tka), tranexamic acid

## Abstract

Background: Tranexamic acid (TXA) is a pharmacological agent used in reducing blood loss during orthopaedic surgeries, including total knee arthroplasty (TKA). Despite its proven efficacy and National Institute for Health and Care Excellence (NICE) guidelines recommending combined topical and intravenous administration, compliance in clinical practice often lags.

Objective: This study aimed to evaluate and improve adherence to NICE guidelines for TXA use during TKA through a quality improvement initiative.

Methods: A two-cycle audit was conducted at Good Hope Hospital, Birmingham, United Kingdom, assessing compliance with TXA guidelines in 93 TKA cases reviewed retrospectively. Educational interventions including presentations, emails, and instructional posters, were implemented between cycles to address non-compliance barriers. Data from operation notes were analyzed, and adherence improvements were evaluated using statistical tests.

Results: The first cycle revealed a 51.2% non-compliance rate. Following interventions, compliance improved significantly, with TXA usage rising from 48.8% to 73.1% (p = 0.016). Combined topical and intravenous administration increased from 7.3% to 30.8% (p= 0.005). Subgroup analyses of the second cycle indicated better compliance in non-tourniquet cases and improved adherence among patients with renal impairment.

Conclusion: Targeted educational interventions significantly improved adherence to TXA guidelines in TKA. Incorporating TXA administration into surgical checklists and further education on its safety, especially in patients with renal impairment, may be helpful in improving compliance.

## Introduction

At the time of tissue injury, both fibrinolysis, as well as the coagulation cascade are activated. The coagulation cascade produces fibrin, whereas fibrinolysis degrades fibrin and reverses its effects [1]. The body maintains a balance between both processes to achieve haemostasis. Tranexamic acid (TXA), first synthesized in 1957, competitively inhibits the activation of plasminogen to plasmin, thereby inhibiting the degradation of fibrin [1]. It also blocks plasmin-induced platelet activation, enabling them to be preserved for clot formation later [2]. Also, as plasmin is associated with a pro-inflammatory response, its inhibition by TXA provides an anti-inflammatory effect [3].

Owing to these benefits, TXA has long been used in many orthopaedic surgeries, including total knee arthroplasties (TKA). It has been shown to significantly reduce the need for blood transfusion in orthopaedic surgeries [4]. The National Institute for Health and Care Excellence (NICE) guidelines recommend combined topical and intravenous administration of Tranexamic acid in patients without renal impairment [5,6]. There is evidence to suggest that dual-route administration maximizes TXA efficacy by addressing both local and systemic bleeding risks and resulting in improved outcomes [7]. We performed a two-cycle audit focussing on adherence to these guidelines in total knee arthroplasties performed at our centre.

## Materials and methods

This quality improvement project was conducted as a two-cycle audit in the Trauma and Orthopaedics Department at Good Hope Hospital, which is a surgical hub for elective hip, knee and shoulder surgery at University Hospitals Birmingham, United Kingdom. The aim of the project was to assess adherence to TXA administration according to NICE guidelines in TKAs performed within our department. Approval was provided by the institutional clinical audits registration committee via CARMS-20567 for the first cycle and via CARMS-21721 for the second cycle. Operation notes for 93 TKA procedures in total (41 cases from the first cycle, and 52 cases from the second audit cycle) were retrospectively reviewed to compare administration practices for TXA before and after the educational intervention aimed at improving adherence to NICE guidelines on the same.

Audit cycles

In the first cycle, electronic records of 41 cases of total knee arthroplasty between 15 September 2023 to 15 December 2023 were retrospectively reviewed. An analysis of the outcomes of the first cycle was followed by an educational intervention to address barriers to compliance with TXA use during TKA. The intervention consisted of departmental education via targeted e-mails to consultants/registrars and the presentation of audit results in a speciality meeting. Posters with information on the administration of TXA and NICE guidelines were pasted on computers all across the elective surgical suite and doctors’ computers. The second audit cycle was conducted on cases operated between 15 February 2024 to 15 May 2024.

Outcome measures

Primary outcome measures were the proportion of cases where TXA was not administered, and the routes of administration of TXA (intravenous, topical or both). Secondary outcome measures were the influence of factors such as renal impairment or tourniquet use on TXA compliance.

Statistical analysis using a two-proportion z-test was performed to determine the significance of differences in compliance rates between both audit cycles. Additionally, descriptive subgroup analyses exploring the impact of tourniquet use and renal impairment on compliance with guidelines were also performed.

## Results

Of the 41 cases in the first audit cycle, TXA was not administered in 21 (51%) total knee arthroplasty cases. Following implementation of educational interventions, this improved in the second cycle with TXA not being administered in only 14 (27%) cases. Correspondingly, in the first cycle, TXA was used in 20 (48.8%) of cases and in the second cycle, it was used in 38 (73.1%) of cases. A two-proportion z-test was conducted to compare the proportions of TXA use between the two cycles. The test yielded a p value of 0.016 implying that the increase in the use of TXA from cycle 1 to cycle 2 was statistically significant. This is depicted in Table 1.

**Table 1:** Comparison of TXA use between first and second audit cycles. TXA: tranexamic acid p = 0.016

Cycle	Total cases	TXA used (%)	TXA Not Used (%)
Cycle 1	41	20 (48.8%)	21 (51.2%)
Cycle 2	52	38 (73.1%)	14 (26.9%)

Combined intravenous and topical TXA was used in 3 (7.3%) cases in the first cycle and this improved to 16 (30.8%) cases in the second cycle (p = 0.005). A detailed description of the mode of administration of TXA is depicted in Table 2.

**Table 2:** Mode of TXA administration between first and second audit cycles. TXA: tranexamic acid p = 0.005

Cycle	Intravenous (%)	Topical (%)	Combined (intravenous + topical) (%)	No TXA (%)	Total
Cycle 1	14 (34.1%)	3 (7.3%)	3 (7.3%)	21 (51.2%)	41
Cycle 2	22 (42.3%)	0 (0%)	16 (30.8%)	14 (26.9%)	52

Impact of tourniquet use

In the first audit cycle, of the 10 patients operated on without using a tourniquet, TXA was not used in five (50%) cases. In cases where a tourniquet was used, TXA was not used in 16 (50%) cases. In the second cycle, we identified a negative correlation between tourniquet use and TXA compliance. When a tourniquet was employed, TXA was not administered in 6 (50%) cases. In contrast, non-compliance was significantly lower at 20% (8 out of 40 patients) when a tourniquet was not used. This is depicted in Table 3 and Table 4.

**Table 3:** TXA compliance with tourniquet use. TXA: tranexamic acid

Cycle	No TXA (%)	Intravenous (%)	Topical (%)	Combined (%)	Total
Cycle 1	16 (51.6%)	11 (35.5%)	2 (6.5%)	2 (6.5%)	31
Cycle 2	6 (50%)	5 (41.6%)	0 (0%)	1 (8.3%)	12

**Table 4:** TXA compliance without tourniquet use. TXA: tranexamic acid

Cycle	No TXA (%)	Intravenous (%)	Topical (%)	Combined (%)	Total
Cycle 1	5 (50%)	3 (30%)	1 (10%)	1 (10%)	10
Cycle 2	8 (20%)	17 (42.5%)	0 (0%)	15 (37.5%)	40

Impact of renal impairment

In the first cycle, there were nine patients with significant renal impairment, defined as an estimated glomerular filtration rate (eGFR) ≤40 mL/min and only one of those patients received 1 g of intravenous tranexamic acid. In the second cycle, there were 12 patients with significant renal impairment. Out of those, six (50%) did not receive TXA. Of those that received, five (41.7%) patients adhered to guidelines and received only 1 g of intravenous TXA. One (8.3%) patient received combined topical plus intravenous TXA but had no untoward effect due to the higher dose received, and their renal function remained stable. This is shown in Table 5.

**Table 5:** Use of TXA in patients with significant renal impairment. TXA: tranexamic acid

Cycle	TXA not used (%)	Intravenous (%)	Combined (intravenous + topical) (%)	Total
Cycle 1	8 (88.9%)	1 (11.1%)	0 (0%)	9
Cycle 2	6 (50%)	5 (4.6%)	1 (8.3%)	12

## Discussion

Despite our centre being a dedicated site for elective hip, knee and shoulder arthroplasty at University Hospitals Birmingham, many hip and knee surgeons were non-compliant with NICE guidelines on the use of tranexamic acid in knee arthroplasties.

The findings from this two-cycle audit highlight the impact of educational interventions in improving adherence to established guidelines. There was a statistically significant improvement in TXA compliance between the first and second audit cycles. This demonstrates that targeted educational measures can effectively address barriers to compliance.

A prospective randomized control trial conducted by Huang et al. noted that patients treated with intravenous and topical TXA had less blood loss, lower post-operative knee swelling, less post-operative pain, better early knee function and better early satisfaction following total knee arthroplasty compared to the same using tourniquet [8]. Also, even in cases where a tourniquet is used, the addition of TXA lowers the total blood loss, risk of blood transfusion, and incidence of pulmonary embolism [9].

In the first audit cycle, TXA was not used in 50% (five out of 10) of cases where surgery was performed without a tourniquet, mirroring the non-compliance rate in cases where a tourniquet was employed (16 out of 31 cases, 50%). However, findings from the second cycle demonstrated that the use of TXA played a pivotal role in enabling surgeries without the use of a tourniquet. When no tourniquet was used, TXA non-compliance dropped significantly to 20% (eight out of 40 cases), compared to a 50% non-compliance rate (six out of 12 cases) when a tourniquet was employed. These results suggest that adherence to TXA guidelines facilitated the avoidance of tourniquet use, adding to the benefits of TXA by avoiding risks associated with tourniquet use. Tourniquet use in TKA is associated with a higher risk of thromboembolic events, higher postoperative pain and prolonged rehabilitation [10]. On the other hand, surgeons performing TKAs using a tourniquet often deflate the tourniquet after performing closure to minimize blood loss [11]. This would prevent topical application as haemostasis would not be an issue while the tourniquet is still inflated. Also, there is evidence to suggest that prolonged tourniquet use may increase the risk of deep-vein thrombosis (DVT), and this imposes a bias against the use of TXA in patients being operated under tourniquet control for fear of increasing the risk of developing DVT [12].

A proposed suggestion for increasing compliance to the use of TXA is incorporating TXA administration into the WHO surgical checklist for routine arthroplasty cases [13]. By including a dedicated checklist item, anaesthetic and surgical staff can reduce omissions due to oversight or variations in practice.

Another barrier to the use of tranexamic acid is its predominantly renal mode of clearance [14]. This might be one of the reasons for apprehension towards administering TXA in patients with renal impairment. Targeted educational interventions should focus on the safety of 1g intravenous TXA even in patients with significant renal impairment (as highlighted by NICE guidelines) to allay unfound fears against the use of TXA in these patients.

Limitations of the study

Limitations of our study are the small sample size and retrospective nature. Also, both cycles conducted within a relatively short timeframe limit the study’s ability to assess the sustainability of improvements or trends in long-term compliance. Future studies could benefit from a larger cohort, which would also make more meaningful subgroup analyses in cases with confounding factors to the use of TXA like renal impairment, use of tourniquet etc. While the study highlighted targeted educational interventions that might increase compliance, it did not include detailed evaluations of why surgeons and anaesthetists deviated from guidelines in the first place. The reasons could be related to concerns over safety, lack of familiarity with NICE guidelines, or other factors not accounted for. This could be an avenue for future research at our institution.

## Conclusions

This audit highlights the potential of educational interventions in enhancing adherence to evidence-based guidelines. By improving TXA administration practices, surgeons can optimize outcomes, reduce blood loss, and promote high-quality care in total knee arthroplasty as per national guidelines.
